# Knowledge about mental illnesses among Tunisian students

**DOI:** 10.1192/j.eurpsy.2023.224

**Published:** 2023-07-19

**Authors:** M. Ben Amor, Y. Zgueb, A. Aissa, U. SchöBerlein Ouali, R. Zaibi Jomli

**Affiliations:** Psychiatry “A”, Razi Hospital, Manouba, Tunisia

## Abstract

**Introduction:**

Mental Health Knowledge specific to symptom recognition, treatment efficacy, help-seeking, and employment can facilitate understanding when communicating with clinicians and reduce personal stigma. Better knowledge of mental illness has also been shown to decrease fear and embarrassment when interacting with people with mental illnesses. Thus, knowledge can play a key role in influencing behaviors and attitudes associated with stigma.

**Objectives:**

The objective of this study was to evaluate mental health knowledge among Tunisian students

**Methods:**

This cross-sectional study was conducted on 2501 Tunisian students from different academic institutions. They anonymously filled in a questionnaire circulated online through social networks in pages and groups of each university. The validated Arabic version of the “Mental Health Knowledge Schedule” (MAKS) was used to assess the knowledge about mental illnesses.

**Results:**

The median MAKS score was equal to 45 out of 60, ranging from 30 to 56. In our study, 60.2% of the participants answered “don’t know” or “neither agree nor disagree” to item 1 indicating that “Most people with mental health problems want to have paid employment.”. Exactly 83.7% of the participants thought they knew what advice to give a friend to get professional help and 90% thought that psychotherapy could be effective in treating a person with a mental illness. In addition, 57.1% of participants thought that medication could be effective and 68.8% thought that people with severe mental health problems could make a full recovery. People with mental health problems do not seek professional help according to 39% of participants. About 90% were considering depression, schizophrenia, and bipolar disorder as mental illnesses. Stress and drug addiction were considered mental illnesses according to 71% and 63% of participants respectively. Finally, 52.9% answered that grief was a mental illness.

**Conclusions:**

In Tunisia, anti-stigma programs are almost nonexistent. Our results would allow us to take a baseline assessment of mental health knowledge and could be the starting point for anti-stigma interventions. We should combine these findings with a behavioral and attitudinal assessment to better address stigma.

**Disclosure of Interest:**

None Declared

